# Arrival time parametric imaging using Sonazoid-enhanced ultrasonography is useful for the detection of spoke-wheel patterns of focal nodular hyperplasia smaller than 3 cm

**DOI:** 10.3892/etm.2013.1048

**Published:** 2013-04-04

**Authors:** NORITAKA WAKUI, RYUJI TAKAYAMA, NAOHISA KAMIYAMA, KOJIRO KOBAYASHI, DAIGO MATSUI, YASUSHI MATSUKIYO, TAKENORI KANEKAWA, TAKASHI IKEHARA, KOJI ISHII, YASUKIYO SUMINO

**Affiliations:** 1 Division of Gastroenterology and Hepatology, Toho University Omori Medical Center, Tokyo 143-8541;; 2 Ultrasound General Imaging, GE Healthcare, Tokyo 191-8503, Japan

**Keywords:** focal nodular hyperplasia, arrival time parametric imaging, micro-flow imaging, Sonazoid, contrast-enhanced ultrasonography, spoke-wheel pattern

## Abstract

It is considered difficult to make a definitive diagnosis of focal nodular hyperplasia (FNH) of <3 cm when using conventional diagnostic imaging modalities. Typical FNH imaging findings are: i) central scar formation, ii) nutrient vessels extending radially from the center and iii) the presence of Kupffer cells. In a clinical setting, identification of a spoke-wheel pattern formed by nutrient vessels extending radially is a key feature in the diagnosis of FNH. In this study, we investigated the detection rate of spoke-wheel patterns of FNH <3 cm using arrival time parametric imaging (At-PI) technology with Sonazoid-enhanced ultrasonography (US). Five patients with FNH <3 cm who had undergone Sonazoid-enhanced US at the Toho University Omori Medical Center between February 2008 and March 2009 were included in the study. The mean tumor diameter was 20.2±7.2 mm. Lesions were enhanced with 0.5 ml Sonazoid US contrast agent and a video of the procedure was saved and used for At-PI analysis of contrast agent dynamics in FNH. Three ultrasonographic specialists examined the images and made a diagnosis of FNH based on the findings of spoke-wheel patterns. Similarly, micro-flow imaging (MFI) was performed to evaluate the contrast agent dynamics in FNH. Using MFI, FNH was diagnosed in 3 of the 5 cases by the three specialists, whereas At-PI enabled the identification of spoke-wheel patterns in all 5 cases. At-PI using Sonazoid-enhanced US is superior for detecting spoke-wheel patterns of FNH <3 cm.

## Introduction

Focal nodular hyperplasia (FNH) is a benign hyperplastic lesion and the number of reports of FNH has been increasing owing to the widespread use of diagnostic imaging at routine medical check-ups. The characteristic imaging findings of FNH are: i) central scar formation, ii) nutrient vessels extending radially from the center and iii) the presence of Kupffer cells (1). When using an imaging modality in the diagnosis of FNH, the key is to identify the spoke-wheel pattern of nutrient vessels radiating from the center of the FNH lesion. However, with contrast-enhanced computed tomography (CT), the detection rate of tumors <3 cm is reportedly as low as 3%, even though the detection rate of tumors >3 cm is 65% (2). With contrast-enhanced ultrasonography (US), the detection rates of tumors >3 cm, <3 cm and <2 cm are 95, 30 and 16.7%, respectively (3). The detection of spoke-wheel patterns in FNH <3 cm is particularly difficult, resulting in low detection rates. Currently, Sonazoid-enhanced US is commonly used to obtain detailed hemodynamic information of hepatic lesions and to make a qualitative diagnosis (4–11); however, due to the monochromatic representation of the contrast agent, detailed visual examination of the hemodynamics in small tumors and tumors with rapid blood flow is often difficult.

In this study, we used arrival time parametric imaging (At-PI), which enables color display of contrast agent dynamics in contrast-enhanced US to investigate the detection rate of spoke-wheel patterns in FNH <3 cm.

## Materials and methods

### Patient background

Five patients (3 males and 2 females) with FNH <3 cm who had undergone Sonazoid-contrast US at the Toho University Omori Medical Center in Tokyo, Japan between February 2008 and March 2009 were enrolled in the study. The mean tumor diameter was 20.2±7.2 mm (Table I). FNH was diagnosed on the basis of high-density signals in the early phase and iso-density signals in the equilibrium phase of abdominal CT as well as histological findings of FNH, including irregular hyperplastic hepatocytes and fibrotic scarring involving abnormal blood vessels in post-US biopsy. This study was performed with approval of the Ethics Committee at Toho University Omori Medical Center. Written informed patient consent was obtained from the patient.

### Sonazoid-enhanced US

Ultrasonography was performed using a Toshiba Aplio XG ultrasound device (SSA-790A; Toshiba Medical Systems, Tochigi, Japan) with a 3.75 MHz convex array probe (PVT-375 BT) at a mechanical index of 0.22–0.29. The focus was set at the bottom end of the tumors and 0.5 ml Sonazoid (perfluorobutane; GE Healthcare, Oslo, Norway) was injected into the cubital vein. Data generated in the first 40 sec was saved as raw data on the hard disk. US was performed by the same operator to maintain imaging consistency.

### At-PI

**Figure 1:**
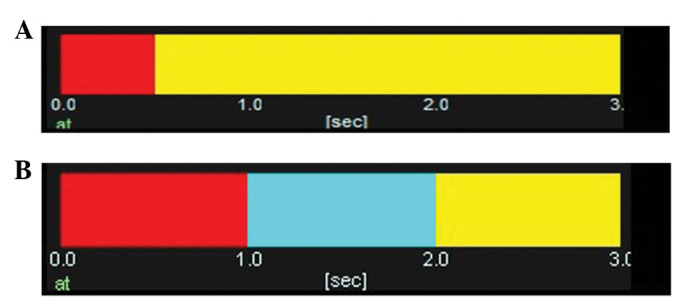
Color scales used in arrival time parametric imaging (At-PI). (A) Parametric-color scale 1 (red, 0–0.5 sec; yellow, >0.5 sec) was used to evaluate lesions contrasted within 2 sec. (B) Parametric-color scale 2 was used for lesions requiring ≥2 sec for contrast enhancement. The scale was generated with red (0–1 sec), cyan (0–2 sec) and yellow (>2 sec).

Following US, At-PI was performed using the proprietary image analysis software of the ultrasound system. The system set the point at which the contrast agent reached the hepatic tumor as time zero and sequentially calculated the arrival time at individual pixels representing the tumor. A color map was created and automatically superimposed on a B-mode image. Then, two parametric-color scales were used to analyze color patterns and the time setting for color distribution. Parametric-color scale 1, with time 0–0.5 sec in red and time >0.5 sec in yellow, was used to analyze tumors contrasted within 2 sec. Parametric-color scale 2 was used for tumors requiring ≥2 sec for contrast enhancement and red, cyan and yellow were used to represent the times 0-1, 1-2 and >2 sec, respectively (Fig. 1).

### Micro-flow imaging

Following US, micro-flow imaging (MFI) was also performed using the proprietary image analysis software of the ultrasound system. The system started counting time when the contrast agent reached the tumor and the arrival time of individual pixels representing the tumor was sequentially calculated. A color map was created and automatically superimposed on a B-mode image.

### Tumor assessment

At-PI video images were evaluated by three ultrasonographic specialists with extensive experience (14, 17 and 33 years) in the contrast-enhanced US of liver diseases. The specialists had no access to the clinical background of the patients, hemanalysis, imaging findings or final diagnosis. Detection of a spoke-wheel pattern was considered a positive imaging finding. Similarly, MFI images were generated to analyze contrast agent dynamics.

## Results

Case 1 (47-year-old female) had a 14-mm tumor with low echo signals in hepatic segment 8 (S8). Since it took 1.7 sec to contrast the entire tumor, parametric-color scale 1 was used in At-PI analysis. Three ultrasonographic specialists identified a spoke-wheel pattern on the At-PI images, even though no identification was made in MFI.

Case 2 (59-year-old male) had a 17-mm tumor with low echo signals in S8. Since 2.5 sec was required to contrast the tumor, parametric-color scale 2 was used in At-PI. Three specialists observed a spoke-wheel pattern in At-PI but not in MFI.

Case 3 (34-year-old male) had a 14-mm tumor with low echo signals in S8. Since 2.8 sec was required for tumor enhancement, parametric-color scale 2 was used in At-PI. A positive diagnosis of FNH was made using MFI and At-PI.

Case 4 (43-year-old female) had a 28-mm tumor with low echo signals in S5. As 2.5 sec was required for tumor enhancement, parametric-color scale 2 was used in the analysis. A positive diagnosis was made using the two imaging modalities.

**Figure 2:**
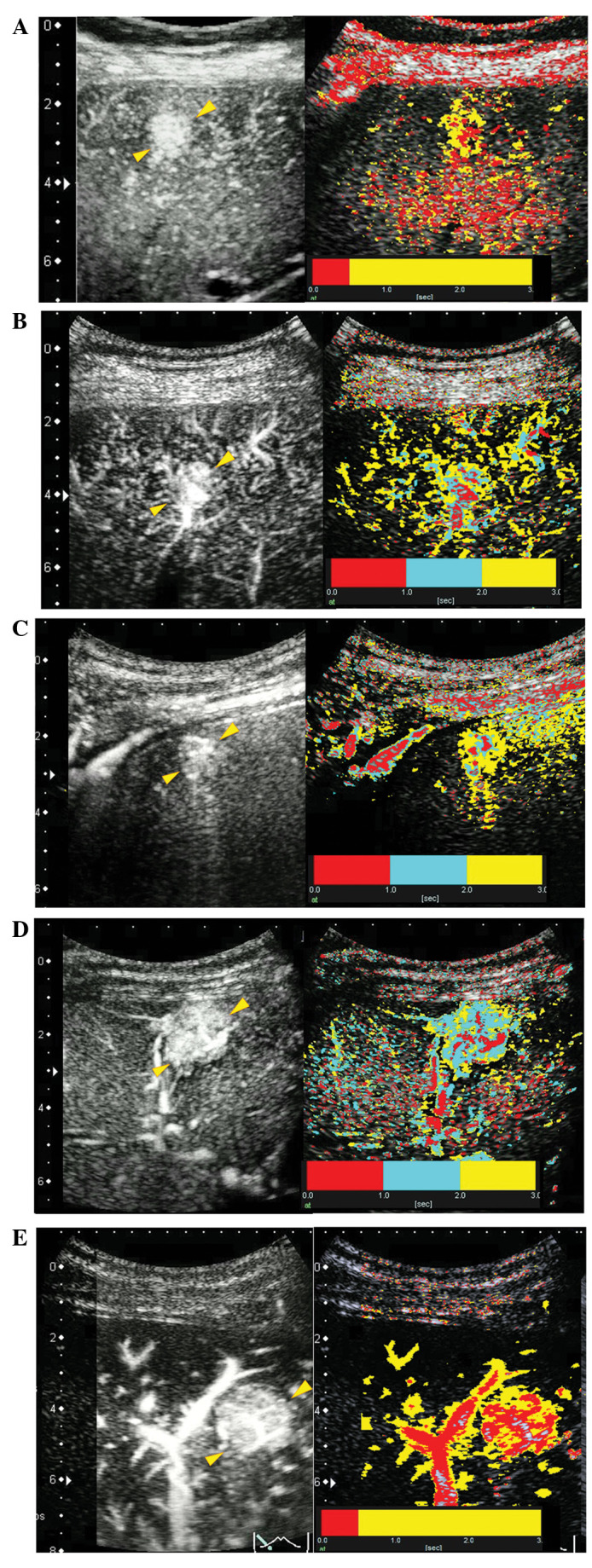
Ultrasound (US) imaging of focal nodular hyperplasia (FNH) in 5 cases. Left, micro-flow imaging (MFI) and right, arrival time parametric imaging (At-PI). In (A) case 1 and (B) case 2, a spoke-wheel pattern was visible in At-PI but not in MFI. In (C-E) cases 3, 4 and 5, respectively, a spoke-wheel pattern was visible in MFI and At-PI. Arrowheads indicate the tumor.

Case 5 (56-year-old male) had a 28-mm tumor with low echo signals in S3. Since it took 2.0 sec to contrast the tumor, parametric-color scale 1 was used in At-PI analysis. A spoke-wheel pattern was identified using MFI and At-PI. Fig. 2 shows the At-PI and MFI images from all cases.

## Discussion

First reported by Edmondson (12) in 1956, FNH is characterized by its high prevalence in females aged 20–50 years (76.2–88%) (13–16) and often exists as a solitary lesion (76.2%) (15,16). Although the pathogenesis of FNH is currently unknown, Wanless *et al* suggested that FNH is caused by hyperplasia of liver parenchyma in response to angiodysplasia since a number of FNH cases have involved vascular and neuroendocrine abnormalities (17,18). An association with birth control pills and thyroid hormones has also been proposed; however, according to certain studies, these agents promote the growth of FNH, but do not cause FNH (19–22).

Typical imaging findings of FNH are: i) central scar formation, ii) nutrient vessels radiating from the center and iii) the presence of Kupffer cells (1). With various forms of diagnostic imaging of FNH, it is essential to identify the spoke-wheel pattern of nutrient vessels radiating from the center of the lesion. Although 95% of FNH >3 cm are detected successfully, the detection of FNH <3 cm is often difficult with a rate of 30% for FNH <3 cm and 16.7% for <2 cm by contrast-enhanced US (3). Since the malignant potential of FNH has been considered negative in previous clonal studies (23–25), patients with a definitive pre-operative diagnosis of FNH are counter-indicated for surgical resection and are generally placed under observation. This also means that a pre-operative needle biopsy is essential in the diagnosis of FNH; however, a definitive diagnosis of FNH <20 mm is often difficult to make (26). Furthermore, the use of needle biopsy in the diagnosis of FNH has been controversial (2,12,27). For this reason, FNH patients are generally followed-up if a diagnosis of FNH is made following comprehensive imaging analysis.

Since its introduction, the ultrasound contrast agent Sonazoid has been used to thoroughly investigate the angioarchitectonic patterns of hepatic lesions whose hemodynamics were previously difficult to understand in detail (4–11). In addition, the MFI function (Toshiba Medical Systems), which was developed to analyze the angioarchitectonic patterns of hepatic lesions, has been reported to be useful (28,29). However, the drawback of MFI is the difficulty associated with the evaluation of angioarchitecture and contrast agent dynamics due to rapid monochromatic enhancement of small lesions or lesions with a short contrast time. By contrast, At-PI has the advantage of displaying temporal changes in contrast-enhanced imaging findings with arbitrary colors, thus having better potential for detecting the spoke-wheel patterns of FNH <3 cm. As anticipated, At-PI enabled us to identify spoke-wheel patterns visually. By contrast, it was difficult with MFI to identify spoke-wheel patterns in 2 of 5 cases, even though MFI, which traces contrast agent dynamics in detail, proved extremely useful in the evaluation of hemodynamics in relatively larger FNH or in FNH that contrasted slowly. In 1 of the 2 failed cases, it took 1.7 sec to contrast the 14-mm lesion and the lesion in another case was 17 mm and took 2.5 sec for contrast enhancement. These tumors were small and contrasted quickly. Even in these situations, the At-PI system enabled the detection of nutrient vessels radiating from the center of tumors due to the arbitrary contrast time settings. To clearly distinguish colors, a longer interval was set to analyze tumors that contrasted slowly, while a shorter interval was set for those that contrasted rapidly and this led to split-second visualization of blood vessels forming a spoke-wheel pattern.

Furthermore, while it is necessary to analyze the video to fully understand contrast agent dynamics in MFI, At-PI enables the diagnosis of FNH by means of a single, static, color-mapped B-mode image that shows the direction and timing of contrast enhancement in the tumor. Therefore, it is easy to explain the findings of contrast agent dynamics, making the system highly useful in a clinical setting. We consider that At-PI is an effective tool for investigating the detailed hemodynamics of small hepatic tumors and tumors with rapid blood flow. At-PI using Sonazoid-enhanced US was useful for identifying spoke-wheel patterns of FNH <3 cm.

## Figures and Tables

**Table I:** Patient characteristics.

Case	Age (years)	Gender (male/female)	Tumor diameter (mm)	Echo level (high/low)	Time needed to contrast an entire tumor (sec)
1	47	F	14	Low	1.7
2	59	M	17	Low	2.5
3	34	M	14	Low	2.8
4	43	F	28	Low	2.5
5	56	M	28	Low	2.0

## Abbreviations:

FNH: focal nodular hyperplasia;
At-PI: arrival time parametric imaging;
MFI: micro-flow imaging;
US: ultrasonography;
CT: computed tomography
